# Metagenome-Assembled Genome Sequences from Different Wastewater Treatment Stages in Germany

**DOI:** 10.1128/MRA.00504-21

**Published:** 2021-07-08

**Authors:** Dominik Schneider, Daniela Zühlke, Anja Poehlein, Katharina Riedel, Rolf Daniel

**Affiliations:** a Department of Genomic and Applied Microbiology, Institute of Microbiology and Genetics, Georg-August University of Göttingen, Göttingen, Germany; b Göttingen Genomics Laboratory, Institute of Microbiology and Genetics, Georg-August University of Göttingen, Göttingen, Germany; c Department of Microbial Physiology and Molecular Biology, Institute of Microbiology, University of Greifswald, Greifswald, Germany; University of Southern California

## Abstract

Metagenome-assembled genome sequences (MAGs) were generated from two wastewater treatment systems in two German cities (Göttingen and Greifswald), based on metagenomes derived from hospital effluent, different wastewater treatment stages, and adjacent water bodies. The MAGs mainly originated from bacterial members of *Proteobacteria*, *Bacteroidota*, *Firmicutes*, “*Candidatus* Patescibacteria,” *Actinobacteriota*, *Chloroflexota*, *Desulfobacterota*, and *Verrucomicrobiota*.

## ANNOUNCEMENT

Municipal wastewater, university hospital wastewater, sludge, and adjacent water bodies at nine and eight locations affiliated with wastewater treatment plants (WWTP) in Göttingen and Greifswald (Germany), respectively, were sampled quarterly (2016 to 2018) (Table S1; https://doi.org/10.6084/m9.figshare.14601126). Three technical replicates from each sample location were collected and processed, and DNA was isolated as described previously (1). Briefly, the planktonic fraction was harvested by centrifugation; the pellets were stabilized with RNAprotect (Qiagen, Hilden, Germany) and stored at 4°C. The RNAprotect was removed and DNA was extracted using the PowerSoil DNA isolation kit (MoBio Laboratories, Inc., Carlsbad, CA, USA). DNA isolations from each sampling site were pooled in equimolar concentrations. The sequencing libraries were constructed and indexed using a Nextera DNA sample preparation kit and an index kit as recommended by the manufacturer (Illumina, San Diego, CA, USA). Paired-end sequencing was performed using a HiSeq 2500 instrument (rapid run mode, 500 cycles) as recommended by the manufacturer (Illumina). Library construction failed for two Bodden samples (March and July 2017), and the sludge was not sampled in 2016 in Greifswald, resulting in 131 metagenomes (Table S1; https://doi.org/10.6084/m9.figshare.14601126).

Default parameters were used for all software unless otherwise specified. R v4.0.2 (2) and RStudio v1.3.1056 (3) were used for data table processing and figure generation. The data processing included fastp v0.20.0 (4) with overlap correction, quality filtering (removal of reads of <Q20), read clipping with a sliding window of 4, the removal of reads shorter than 50 bp, and Illumina adapter removal. After quality filtering, the metagenome sequences consisted of 5.7 billion paired-end reads with an average read length of 207 bp (forward) and 206 bp (reverse), respectively (Table S1; https://doi.org/10.6084/m9.figshare.14601126).

The samples were merged by site and city, resulting in 17 data sets, which were assembled using metaSPAdes v3.13.0 (5) with defined kmers (-k 21,33,55,77,99,127) and without error correction (- -only-assembler). Contigs with lengths of <1,000 bp were discarded using USEARCH v9.2.64 (6). The assembly characteristics were calculated using BBMap’s statswrapper.sh (https://sourceforge.net/projects/bbmap/) and are summarized in Table S2 (https://doi.org/10.6084/m9.figshare.14601129).

The contig coverage information was determined using Bowtie2 v2.3.5.1 (7) and SAMtools v1.9 (8). Metagenome-assembled genome sequences (MAGs) were generated using MetaBAT2 v2.12.1 (9). The MAG quality and average coverage were determined using CheckM v1.1.2 (10). The MAG bins were classified as high, medium, and low quality according to minimum information MAGs (MIMAGs) (11). The rRNA and tRNA genes were annotated using Prokka v1.14.5 (12). In addition, MAG bins with <10-fold coverage, <500 kbp, <20% completeness, and >1,000 contigs were removed. The overall average sequencing depth was 44-fold. The MAGs were classified taxonomically using GTDB-Tk v1.3.0 (13) and the Genome Taxonomy Database (GTDB) r95 (14).

**FIG 1:**
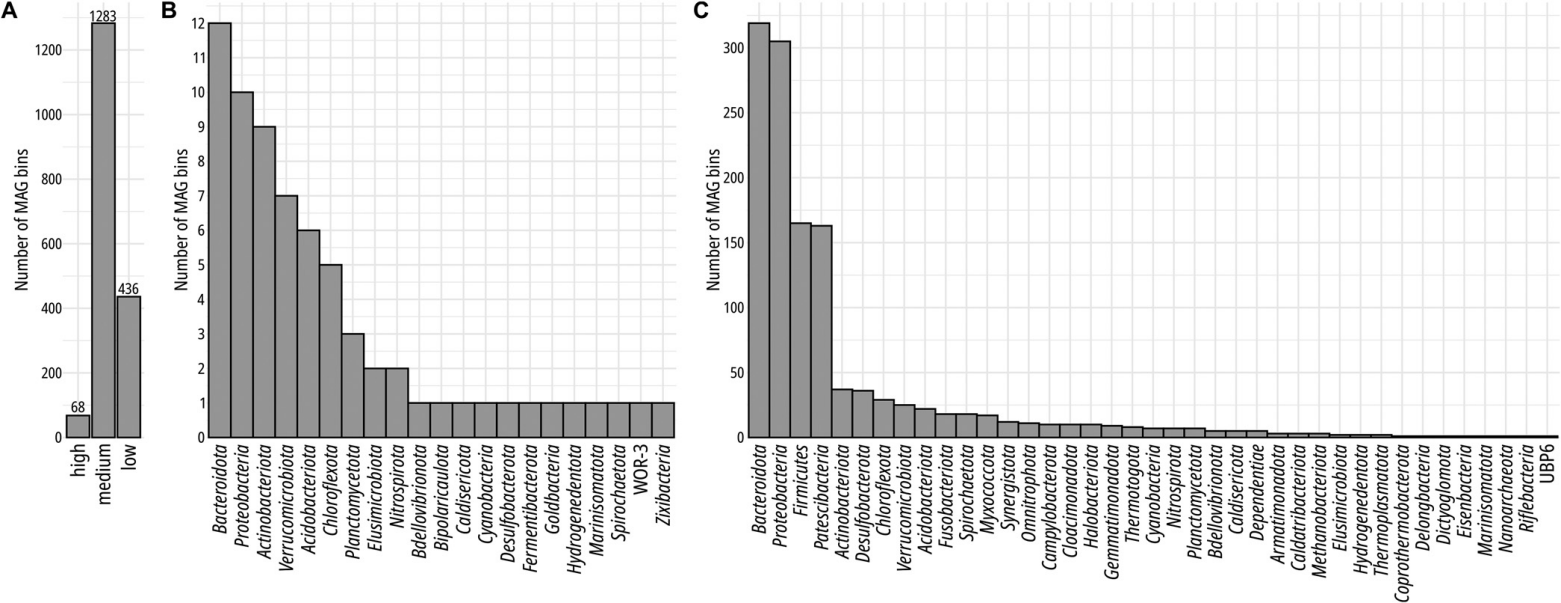
Overview of all generated MAGs from two wastewater treatment systems in Göttingen and Greifswald. (A) Number of MAG bins with high, medium, and low quality according to MIMAG. (B and C) Phylum distribution of MAGs of high (B) and medium (C) quality.

This resulted in 68 high-, 1,283 medium-, and 436 low-quality MAGs (Fig. 1), which belong to *Archaea* (21 MAGs) and *Bacteria* (1,766 MAGs). All high-quality MAGs were of bacterial origin and comprised *Bacteroidota* (*Bacteroidia*, 9), *Proteobacteria* (*Alphaproteobacteria*, 5; *Gammaproteobacteria*, 3), *Verrucomicrobiota* (*Verrucomicrobiae*, 5; *Kiritimatiellae*, 1), *Acidobacteriota* (*Aminicenantia*, 1; *Thermoanaerobaculia*, 2; “*Candidatus* UBA6911,” 1; *Vicinamibacteria*, 1), *Actinobacteriota* (*Acidimicrobiia*, 3; *Actinomycetia*, 1), *Planctomycetota* (*Phycisphaerae*, 2; “*Candidatus* UBA8108,” 1), *Chloroflexota* (*Anaerolineae*, 2), *Elusimicrobiota* (*Elusimicrobia*, 2), *Bdellovibrionota* (“*Candidatus* UBA2394,” 1), “*Candidatus* Bipolaricaulota” (*Bipolaricaulia*, 1), *Caldisericota* (*Caldisericia*, 1), *Cyanobacteria* (*Vampirovibrionia*, 1), *Desulfobacterota* (*Syntrophia*, 1), *Fermentibacterota* (“*Candidatus* Fermentibacteria,” 1), “*Candidatus* Goldbacteria” (“*Candidatus* PGYV01,” 1), *Hydrogenedentota* (*Hydrogenedentia*, 1), *Marinisomatota* (“*Candidatus* UBA2242,” 1), *Nitrospirota* (*Nitrospiria*, 1), *Spirochaetota* (“*Candidatus* UBA4802,” 1), “*Candidatus* WOR-3” (“*Candidatus* Hydrothermia,” 1), and “*Candidatus* Zixibacteria” (“*Candidatus* MSB-5A5,” 1). Details for all generated MAGs are provided in Table S3 (https://doi.org/10.6084/m9.figshare.14601141).

### Data availability.

The raw sequences of the metagenomes have been deposited in the NCBI Sequence Read Archive under the BioProject accession number PRJNA524094; details are listed in Table S1 (https://doi.org/10.6084/m9.figshare.14601126). The coassembled metagenomes (https://doi.org/10.6084/m9.figshare.14578308) and MAGs (https://doi.org/10.6084/m9.figshare.14578629) are also available.
